# 2223. Evaluation of the Microbiology and Clinical Outcomes of Spontaneous Bacterial Peritonitis (SBP) within a US Academic Health System

**DOI:** 10.1093/ofid/ofad500.1845

**Published:** 2023-11-27

**Authors:** P Chris Parish, Alex D Taylor, Tyler J Stone, Vera Luther, Christopher Ohl, James R Beardsley

**Affiliations:** Atrium Health Wake Forest Baptist, Winston-Salem, North Carolina; Atrium Health Wake Forest Baptist, Winston-Salem, North Carolina; Wake Forest Baptist Health, Winston-Salem, North Carolina; Wake Forest University School of Medicine, Winston Salem, NC; Wake Forest School of Medicine, Winston Salem, North Carolina; Wake Forest University School of Medicine, Winston Salem, NC

## Abstract

**Background:**

2021 SBP treatment guidelines recommend empiric therapy with spectra broader than 3^rd^ generation cephalosporins (TGC) in patients (pts) at risk for multidrug-resistant organisms (MDOs), including those admitted to ICUs, with nosocomial infections, or with recent hospitalization. This study aimed to characterize the microbiology and clinical outcomes of SBP in our health system to determine the applicability of these guidelines.

**Methods:**

Setting: 5-hospital academic health system with > 1500 beds. Design: retrospective, observational study. Adult pts from 2010-2022 with ICD 9/10 codes for SBP & liver dysfunction were screened. Pts with concomitant secondary peritonitis were excluded. Study included all pts with culture-positive (CX-pos) SBP and randomly screened pts with culture-negative (CX-neg) SBP to provide a total sample of 80 pts. Primary outcome was % of peritoneal fluid CX with organisms non-susceptible to ceftriaxone (CRO) in CX-pos pts. Secondary outcomes included mortality & effect of MDO risk factors (hospitalization in prior 90 days, ICU admission, or nosocomial infection) on microbiological & clinical outcomes.

**Results:**

49 pts with CX-pos and 31 pts with CX-neg SBP were included. Median age was 59; median MELD score was 25; 26 (33%) were admitted to the ICU. 67 of 80 (84%) total pts had ≥ 1 MDO risk factor; 40 of 49 (82%) in the CX-pos cohort. 8 (16%) isolates were CRO-non-susceptible, 7 in the 40 CX-pos pts with MDO risk factors (17.5%) vs. 1 in the 9 CX-pos pts without risk factors (11%) (p=NS). 49 (61%) pts received initial TGC therapy. Mortality at 30 days & 90 days was 29 (43%) & 40 (60%) and 3 (23%) & 6 (46%) for pts with and without MDO risk factors, respectively. Outcomes based on empiric therapy are summarized in Table 1.

**Figure:**
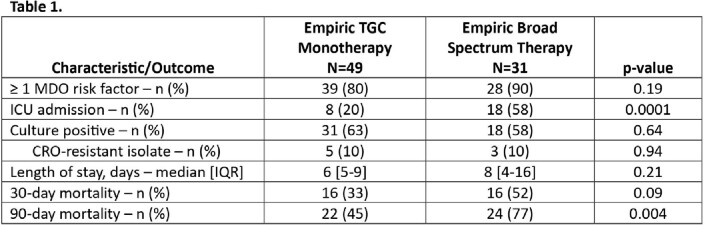

**Conclusion:**

In our facilities, 16% of isolates in CX-pos SBP pts were not susceptible to CRO. There was no significant difference in the number of non-susceptible isolates in pts with and without MDO risk factors. Treatment with broader therapy was not associated with improved outcomes. Within our health system, giving all SBP pts with MDO risk factors broad empiric therapy may not be necessary. Evaluating local microbiologic data may help health systems assess the local applicability of national treatment guidelines.

**Disclosures:**

**Tyler J. Stone, PharmD**, ViiV Healthcare: Employee

